# The burden of unexpected costs in medical school

**DOI:** 10.1371/journal.pone.0312401

**Published:** 2024-12-05

**Authors:** Madeleine Ball, LeAnn Lam, Megan Tigue, Simone Herzberg, Luke Finck

**Affiliations:** 1 University of California, San Francisco, San Francisco, California, United States of America; 2 Massachusetts General Hospital, Boston, Massachusetts, United States of America; 3 Vanderbilt University School of Medicine, Nashville, Tennessee, United States of America; Woldia University, ETHIOPIA

## Abstract

**Background:**

The cost of medical school continues to climb. What has yet to be well-understood are the so-called “unexpected costs” of medical school that, while not explicitly required, are considered paramount to success. This study aims to identify, quantify, and analyze the impacts of these costs of medical school and offer suggestions in alleviating these burdens.

**Methods:**

Medical students across the United States were administered a questionnaire inquiring about the unexpected costs of medical school including test prep materials, clinical supplies, student fees, and the impact of these costs on mental and financial health. In this multi-institutional, cross-sectional survey study, outcomes included total cost, perception of expected vs. actual cost, and impact on well-being.

**Results:**

From January to June 2022, 499 survey responses were collected. The average total additional costs were $4,937, with 83% of respondents stating they spent more during medical school than expected. 51% reported their institutions’ cost of attendance did not accurately predict expenses. These costs were a financial constraint for 68% of students, with a significant increase in constraint for first-generation college students (89% vs 65%, p = 0.02). Financial concerns impacted 65% of students’ mental health and well-being during their medical career, with a significantly increased impact on first-generation college students (85% vs 62%, p = 0.045).

**Conclusions:**

Most students believed the total cost of medical school was significantly more than expected, serving as a source of stress and negatively impacting their mental health. The financial constraints and impact on mental health and well-being were most pronounced for first-generation college students. This study illuminates the importance of improving communication regarding costs outside of tuition and providing support and advocacy to alleviate those costs. Future investigation should further examine how other demographics are impacted by the burden of unexpected costs in medical school.

## Introduction

According to the AAMC’s Tuition and Student Fees Reports, the average cost of tuition, fees, and health insurance at U.S. medical schools for the 2021–2022 school year ranges from $38,947 for in-state residents at public universities to $62,539 for private medical schools [1]. However, this figure does not represent all costs incurred by medical students. The true cost of attendance for medical students including textbooks, housing, transportation, food, and other costs of living, varies greatly by location and is often prohibitively expensive [2]. Most notably, these include paid subscriptions to outside resources such as online board examination practice questions, video content, and other applications. Each year students discover that, while not explicitly required, these subscriptions are largely considered paramount to success. The extent and impact of these so-called “unexpected costs” are not well understood.

Importantly, as the reported cost of attendance (COA) is compounded by these rising costs, the financial burden placed on our medical students is becoming prohibitively expensive. As this financial burden continues to compound, the majority of medical students are forced to take out loans. Nearly 81% of M.D. and D.O. graduates, graduated with student loan debt that averaged $223,000 in 2016 [3]. Issues stemming from rising medical debt include influence on specialty decision and disproportionately affect students from low-income and ethnic/minority backgrounds [4–6]. In a recent survey of medical school applicants, the cost of attending medical school was identified as one of the top reasons for not applying to medical school [7]. With rapidly rising rates of medical student debt, the costs versus value of medical education is a topic that has begun to garner interest [3, 4, 6, 8].

A recent New York Times article described the emotional and physical toll of this dilemma, particularly on low-income and under-represented minority students [4]. Using stories from current medical students, it describes in detail several of these unexpected costs from MCAT prep materials to USMLE board examination fees. There have been numerous studies focused on the cost of applying to medical school as well as solutions to decrease these rising costs [3, 5, 7]. However, no formal study has quantified the total cost, expected and unexpected, incurred during medical school, nor determined its impact on student success and well-being.

Through surveys disseminated to medical schools throughout the country, this study aims to identify, quantify, and analyze the impacts of these unexpected costs of medical school and offer suggestions for alleviating these burdens.

## Materials and methods

### Study design and participants

This is a multi-institutional, cross-sectional survey study performed from 2021 to 2022. Study institutions included U.S. DO and MD-granting schools in the United States, representing diversity in geography and public/private status to address concerns of coverage bias. Medical students with English as their primary language were included in this analysis. At the time of survey completion, participating students were current medical students and MD-PhD students. This study was approved and deemed exempt by the Vanderbilt University IRB (IRB #202463). Informed consent was deemed unnecessary as surveys were anonymous, with no personally identifying information collected. Also, IRB approval from other participating schools was deemed unnecessary because all primary authors with access to data were affiliated with Vanderbilt.

### Survey instrument

Survey items focused on four main categories: (1) background information about the student, the school, and student financial support, (2) estimates of unexpected costs; (3) impact on financial and mental wellbeing and (4) demographics. Survey responses took an average of 5 minutes to complete. Medical students, experienced medical educators, and researchers developed these items through a series of virtual and in-person meetings and literature review of costs in medical education.

Items quantifying costs for various resources were rounded to the nearest whole dollar with a maximum of $5000+ for all items except residency fees (maximum increased to $15,000+). Items assessing impact were rated on a 5-point Likert scale (ranging from strongly disagree to strongly agree). To address concerns of survey validity, pilot surveys were tested in small groups, with subsequent focus groups of participants. Modifications and revisions were included prior to final survey distribution. The survey instrument is available in S1 Fig.

### Data collection

A REDCap survey was distributed to medical students via email with up to one follow-up reminder. The survey was distributed both via the American Association of Medical Colleges (AAMC) listserv [9], as well as through student-wide emails at seven representative medical schools. The AAMC listserv was selected for distribution to include representation of medical schools throughout the country, including diversity of region and institution type. The email indicated that participation was voluntary and responses were anonymous. Survey responses were collected from January 25, 2022 to June 29, 2022 and the demographics of our surveyed population approximated that of all U.S. medical schools, according to AAMC data from the 2018–19 school year [10].

### Data analysis

Analyses were performed using STATA statistical software, version Stata/MP 16.1 for Mac (Intel 64-bit) (College Station, TX). Categorical data was represented using summary statistics (Mean and Standard Deviation). P-values for all categorical variables were calculated using a Fisher Exact Test at an alpha of 0.05.

## Results

Survey responses were collected from January 2022 to June 2022. 499 responses were received and included in analysis. 63% of respondents attended private MD institutions and 37% attended public MD institutions, evenly distributed throughout class years (Table 1). 13% were first-generation college students and 78% were first-generation medical students. All other demographic information is reported in Table 1. The mean total estimated debt upon graduation was $153,000 (SD 121.63). First-generation college or medical students reported an average total debt of $190,000 (SD 98.75) (Table 2).

**Table 1:** Survey respondent demographics.

Characteristic	No (%) of respondents (n = 499)
**Institution Type**	
** Private MD-granting institution**	63.13% (n = 315)
** Public MD-granting institution**	36.67% (n = 183)
** Private DO-granting institution**	0.20% (n = 1)
**Year of Training**	
** M1 **	18.27% (n = 91)
** M2 **	24.10% (n = 120)
** M3**	28.71% (n = 143)
** M4**	24.30% (n = 121)
** Graduate, research, other year out**	4.42% (n = 22)
** Other**	0.20% (n = 1)
**Age**	
** Mean**	25.77
** Standard Deviation**	2.56
** Min**	21
** Max**	43
**Gender Identity**	
** Woman**	63.38% (n = 315)
** Man**	34.00% (n = 169)
** Non-binary/non-conforming**	1.81% (n = 9)
** Unknown**	0.80% (n = 4)
**Race**	
** American Indian or Alaska Native**	0.41% (n = 2)
** Asian**	20.28% (n = 100)
** Black or African American**	9.94% (n = 49)
** Native Hawaiian or Pacific Islander**	0.41% (n = 2)
** White**	62.47% (n = 208)
** Unknown**	6.49% (n = 32)
**Hispanic, Latinx, or Spanish origin**	
** Yes**	10.53% (n = 52)
** No**	88.06% (n = 435)
** Unknown**	1.42% (n = 7)
**First-Generation College Student**	
** Yes**	12.83% (n = 64)
** No**	85.97% (n = 429)
** Unsure**	0.80% (n = 4)
** Unknown**	0.40% (n = 2)
**First-Generation Medical Student**	
** Yes**	78.07% (n = 388)
** No**	21.13% (n = 105)
** Unsure**	0.40% (n = 2)
** Unknown**	0.40% (n = 2)
**Tuition and Scholarships**	
** None**	30.26% (n = 151)
** MSTP**	7.01% (n = 35)
** Full scholarship**	14.83% (n = 74)
** Partial scholarship**	47.49% (n = 237)
** Prefer not to answer**	0.40% (n = 2)

**Table 2:** Self-reported estimated debt upon graduation.

Estimated Debt	Mean $ (S.D.)
**Total**	152,760 (5,506)
**M1 respondents**	156,900 (12,560)
**M2 respondents**	141,060 (11,821)
**M3 respondents**	161,640 (9,621)
**M4 respondents**	165,680 (11,281)
**Graduate student**	15,380 (8,435)
**Research, professional, other year out **	188,000 (45,534)
**Respondents with any scholarship**	17,940 (6,770)
**First-generation college or medical students**	190,430 (12,344)

Table 3 summarizes the out-of-pocket unexpected expenses paid by medical students, including items necessary for clinical duties, test preparation materials and textbooks, and the costs of required board exams. One hundred percent of students surveyed reported paying for out-of-pocket expenses beyond tuition and fees paid directly to their schools. The average sum of additional costs beyond tuition was $4,937 (Table 3). There were no significant differences in these expenses based on public versus private education, age, race, or first-generation status (data not shown).

**Table 3:** Total unexpected costs by medical school year of training.

	Total	M1	M2	M3	M4	Other
	*Mean $*	*Mean $ *	*Mean $*	*Mean $ *	*Mean $ *	*Mean $ *
	* (SD)*	*(SD)*	*(SD)*	*(SD)*	*(SD)*	*(SD)*
**Clinical supplies **	544.12 (30.84)	458.52 (67.94)	559.21 (67.64)	559.22 (57.21)	583.33 (63.55)	506.82 (121.02)
**Test prep and 3rd party resources **	1345.01 (48.48)	459.30 (82.41)	939.92 (74.68)	1732.98 (85.50)	1915.70 (92.25)	1716.67 (215.75)
**Clothing and attire**	598.48 (24.51)	435.39 (50.29)	525.00 (42.15)	625.00 (40.03)	727.08 (61.24)	786.36 (207.17)
**Textbooks **	203.89 (18.53)	142.61 (41.84)	123.50 (19.71)	218.09 (28.82)	306.72 (54.76)	211.36 (65.46)
**Required board examinations **	1155.65 (38.17)	357.14 (111.60)	667.02 (69.00)	1352.88 (46.04)	1629.75 (59.97)	1363.64 (126.35)
**Social/student fees **	873.96 (48.65)	766.09 (80.98)	699.57 (91.22)	901.79 (95.77)	1104.31 (118.67)	809.52 (160.71)
**Residency fees **	1364.61 (245.495)	N/A	N/A	N/A	1891.38 (283.13)	1575.00 (1208.56)
**Total**	4937.16 (348.74)	2875.00 (736.90)	2086.36 (418.85)	5983.33 (1406.48)	6160.49 (459.89)	5275.00 (1694.41)

A total of 83% of respondents reported spending more money out of pocket during medical school than expected, with 51% reporting that their institutions’ cost of attendance did not accurately predict expenses during medical school (Table 4). The cost of additional resources in medical school was a financial constraint for 68% of students, with a significant increase for first-generation college students (89% vs 65%, p = 0.02). Financial concerns impacted 65% of students’ mental health and well-being during their medical career, again with a significantly increased impact on first-generation college students (85% vs 62%, p = 0.045) (Table 5). The impact of unexpected costs by all studied demographics can be viewed in Tables 4–7.

**Table 4:** Impact of unexpected costs during medical school by year of training.

	Total	M1	M2	M3	M4	Other	p-value
**I spent more money out of pocket during medical school than expected**	82.86%	71.42%	83.58%	88.13%	85.71%	100%	0.21
**The total cost of attendance listed by my medical school accurately predicted my expenses during medical school**	48.73%	58.49%	48.48%	50.64%	40.29%	60%	0.44
**The cost of additional resources has been a financial constraint at some point in my medical career **	67.70%	61.36%	65.65%	75.36%	66.67%	66.67%	0.71
**Financial concerns have impacted my mental health and well-being at some point in my medical career**	65.02%	72.27%	70.59%	62.16%	56.14%	50.00%	0.409

**Table 5:** Impact of unexpected costs during medical school by generational status.

	First-Generation College Student	Not First-Generation College Student	p-value
**I spent more money out of pocket during medical school than expected**	96.55%	80.84%	0.166
**The total cost of attendance listed by my medical school accurately predicted my expenses during medical school**	49.15%	44.73%	0.726
**The cost of additional resources has been a financial constraint at some point in my medical career **	88.89%	64.76%	0.02*
**Financial concerns have impacted my mental health and well-being at some point in my medical career**	84.62%	62.29%	0.045*

**Table 6:** Impact of unexpected costs during medical school by race.

	Asian	Black	White	Other	p-value
**I spent more money out of pocket during medical school than expected**	80.00%	88.23%	82.23%	90.91%	0.76
**The total cost of attendance listed by my medical school accurately predicted my expenses during medical school**	51.79%	44.44%	49.40%	45.00%	0.674
**The cost of additional resources has been a financial constraint at some point in my medical career **	60.42%	72.73%	67.46%	92.30%	0.376
**Financial concerns have impacted my mental health and well-being at some point in my medical career**	62.71%	68.18%	63.69%	83.33%	0.69

**Table 7:** Impact of unexpected costs during medical school by school type.

	Private	Public	p-value
**I spent more money out of pocket during medical school than expected**	82.04%	84.81%	0.292
**The total cost of attendance listed by my medical school accurately predicted my expenses during medical school**	51.70%	43.43%	0.188
**The cost of additional resources has been a financial constraint at some point in my medical career **	64.45%	73.63%	0.133
**Financial concerns have impacted my mental health and well-being at some point in my medical career**	63.01%	68.13%	0.407

## Discussion

The costs of applying to and attending medical school are significant and represent a barrier for many into the field of medicine [3, 6]. On top of tuition and the expenses laid out by medical school financial aid offices and registrars, there are numerous additional costs that students are forced to take on. In this multi-institutional, cross-sectional study, we describe the current landscape of unexpected medical school costs and the impacts of these costs on students. Our study is the first, to our knowledge, that quantifies these additional costs, which are essentially universal but often unknown to students prior to matriculation. These results offer a novel target to alleviate the rising burden of debt in medical education. While some institutions may include portions of these costs in their total cost of attendance, the information that is disseminated is not uniform across programs. The overwhelming majority of students (83%) in our survey of 499 students across the United States reported that their expenses during medical school were higher than anticipated. Our study indicates that the costs of these outside resources and subscriptions likely play a significant role in these deviating expectations.

Financial strain and debt burden are common causes of stress among medical students and residents and can lead to burnout, specialty decision, professional impairment, depression, and more [11–13]. As costs continue to rise, the threat of a “bubble and crash” phenomenon in medical education has been proposed, whereby medical school debt to income ratio becomes so high that the prospect of entering medical school is no longer attractive, further increasing the physician shortage [14]. As expected, most students surveyed agreed that the cost of additional resources was a financial constraint for them at some point during their medical school career. Concerningly, these financial constraints impact the mental health and well-being of the majority of students, in-line with previous studies that found a negative association between debt and mental well-being [13]. Interestingly, in our study, first generation college students were disproportionately adversely affected. There were no differences in the perceptions of these unexpected costs between students with and without full or partial scholarships. These results illuminate the undue burden these unexpected costs of medical education place on medical students, particularly those who are the first in their family to pursue higher education. Further research is needed to understand the full implications that these costs have on student health and well-being, particularly in this demographic of students.

How these additional costs impact recruitment and retainment of racially diverse medical students is also not yet clear. Overall levels of debt associated with medical education differ by race and ethnicity. Studies of self-reported debt demonstrate that medical students from populations underrepresented in medicine, particularly those who identify as Black, are more likely to have educational debt and have higher median education-associated debt ($230,000 for Black students vs $200,000 for all students) [15, 16], data that will become increasingly relevant as medical schools continue to become more diverse [17]. While our study did not show any statistically significant differences in the financial or mental-health impacts of these expenses based on race or ethnicity, the overall percentage of students reporting adverse impacts of these costs was so high, there may not have been enough respondents who were unaffected to discern a difference by race. For example, though no statistical significance was identified, Black students reported a relative increased impact of these costs across all categories. Black students reported spending more on medical school than expected (88% vs. 80% of Asian American and 82% of White students), inaccurate costs of attendance (56% vs. 48% and 51%), increased financial constraint of resources (73% vs. 60% and 67%), and increased impact on mental health (68% vs. 63% and 64%). Future studies should further investigate the impact of these costs by race.

Some limitations of our study include the type of institutions sampled, with only one student from DO granting programs surveyed and only 37% of respondents being from public medical schools. Otherwise, the demographics of our surveyed population approximated that of all U.S. medical schools, according to AAMC data from the 2018–19 school year, with few exceptions [10]. Women made up 63% of our survey respondents compared to 56% of all U.S. medical schools. By race, American Indian or Alaska Native made up 0.41% of our survey compared to 0.2% of the national average, 20% were Asian compared to 22%, 10% were Black compared to 6%, 0.4% were Native Hawaiian or Pacific Islander compared to 0.1%, 62% were white compared to 55%, and 10% were Hispanic or Latinx compared to 5.3% [10]. Further, as with all self-report studies, our study may be limited by recall bias, as individuals may have misremembered exact costs when reporting estimates. To combat this, we were careful in question selection, asked respondents to recall a relatively short period of time, and used a focus group prior to survey distribution. Given our study included variation in locations surveyed, our study reflects an average across a wide range of regional costs. As such, this estimate may be higher or lower depending on regional cost variation. Finally, this study was conducted during the transition from in-person to remote residency interviews as well as the modification of USMLE Step 1 to pass/fail grading. In the future, an analysis of how these costs have changed in light of these important process modifications would be interesting.

A key strength of this study is the novel nature of this data. Current literature has offered potential solutions to decreasing the rising cost of medical school, such as reducing medical school tuition, decreasing medical school and residency duration, increasing residency compensation, and developing salary-specific payment plans [7, 18]. To our knowledge, however, no study has investigated the impact of unexpected costs of medical education nor posited solutions for their alleviation. We believe that transparency in quantifying and disclosing these “unexpected” costs is key to combating their effects on student well-being. Given that 83% of our survey respondents reported paying more out-of-pocket expenses than they expected, it is reasonable to suggest that medical schools could provide better estimates of total costs to matriculating students. Perhaps by using a survey tool similar to our own, they could better estimate how much students spend on a yearly basis on study resources, housing, etc. at their individual institutions.

While providing more accurate expectations of costs is important, mitigating the financial burden imposed by some of these costs is critical to improving student well-being. Some institutions provide access to subscription study services or partner with companies to offer discounts to students at their institutions. Assuming this does not lead to a rise in tuition rates overall, this solution could be beneficial to students at these institutions; however, continued communication with students about the efficacy of this approach should occur concurrently. Outside of the realm of medical schools themselves, test-prep companies, scrub companies, and providers of other crucial clinical supplies, could offer need-based financial support or significant discounts for students. This would likely require advocacy and/or financial contributions from medical institutions or larger medical associations. UWorld, a national leader in high-stakes exam preparation including for medical licensing exams, currently offers scholarships for bar, CPA, and CFA exam preparation materials [19]. Expanding this to include USMLE preparation materials could positively impact medical students with the greatest need, yet still may not benefit the majority of students. More research and work need to be done to determine how best to support the broader medical student community financially and relieve the burden of these additional costs.

## Conclusions

These findings underscore the need for increased transparency of costs, improved dissemination of financial aid resources, and methods to alleviate the costs of additional resources outside of tuition alone. Strong and united advocacy for cost reduction by 3rd-party resource providers would be a necessary step toward meaningful improvement of this situation. Future studies should further consider how demographic and socioeconomic factors impact debt and perceptions of unexpected expenses during medical school and training. Ultimately, appropriate management of this challenge will require cooperation between an interdisciplinary group of third-party companies, educators, and institutional leadership.

## Supporting information

S1 Fig Complete survey instrument. This supplement shows the complete survey instrument distributed to medical schools across the country including all items across four main categories: (1) background information about the student and school, (2) estimates of unexpected costs; (3) impact on financial and mental wellbeing and (4) demographics.
(PDF)
