# Supplementary material for: The burden of unexpected costs in medical school
Source: PLoS One. 2024 Dec 5;19(12):e0312401. doi: 10.1371/journal.pone.0312401 (PMC11620594; doi:10.1371/journal.pone.0312401)
Supplement: S1 Fig — This supplement shows the complete survey instrument distributed to medical schools across the country including all items across four main categories: (1) background information about the student and school, (2) estimates of unexpected costs; (3) impact on financial and mental wellbeing and (4) demographics. (PDF) [file pone.0312401.s001.pdf]

Background information

School type

- ◯ Public MD
- ◯ Private MD
- ◯ Public DO
- ◯ Private DO

What is your class year?

- ◯ M1
- ◯ M2
- ◯ M3
- ◯ M4
- ◯ Graduate student, professional student years
- ◯ Research/Year out
- ◯ Other

If other, please describe

____

Are you a first generation college student? (i.e. first of immediate family members to graduate from college)

- ◯ Yes
- ◯ No
- ◯ Unsure
- ◯ Prefer not to answer

Are you a first generation medical student? (i.e. first of immediate family members to graduate from medical school)

- ◯ Yes
- ◯ No
- ◯ Unsure
- ◯ Prefer not to answer

Do you receive financial support from parents, grandparents, etc.? (tuition, rent, monthly allowance, groceries, etc).

- ◯ Yes
- ◯ No
- ◯ Prefer not to answer

What is your current household income? This includes any financial support during medical school from spouses, parents, grandparents, caregivers, etc.

$0K — $400K +

Change the slider above to set a response

reset

What is your estimated debt after graduating medical school? Please include all anticipated debt.

$0K — $400K +

Change the slider above to set a response

reset

Are you receiving a tuition scholarship?

- ◯ Yes, full
- ◯ Yes, partial
- ◯ Yes, MSTP or other PhD funded program
- ◯ No
- ◯ Prefer not to answer

Unexpected Costs
Think about costs that are NOT required and not necessarily listed in estimated cost of attendance - things suggested by peers, faculty, advisors, etc.

To the best of your abilities, please estimate the following:

How much you spent on supplies/materials for clinical duties (ex: stethoscope, ophthalmoscope, reflex hammer, pen light, tuning fork, suture kits, tablet)

$0 — $5000+

Change the slider above to set a response

reset

How much you spent on test prep/3rd party resources (ex: UWorld, Amboss, Sketchy, First Aid, Boards and Beyond, Pathoma, Osmosis, anatomy subscriptions, NBME practice tests)

$0 — $5000+

Change the slider above to set a response

reset

How much you spent on clothing and attire appropriate for the hospital/clinic (business outfits, dress shoes, haircuts)

$0 — $5000+

Change the slider above to set a response

reset

How much you spent on textbooks for preclinical and clinical duties (including online copies of textbooks)

$0 — $5000+

Change the slider above to set a response

reset

How much you spent on required board exams (Step 1, 2)

$0 — $5000+

Change the slider above to set a response

reset

How much you spent on social/student fees (social activities/outings, school clothes and gear, student fees)

$0 — $5000+

Change the slider above to set a response

reset

How much you spent on residency fees (travel, hotel, application fees, background check fees). If this does not apply to you, leave it blank.

$0 — $15000+

Change the slider above to set a response

reset

Please mark the items that were required/included in the estimated costs given to you by your school.

- ☐ Supplies/materials for clinical duties
- ☐ Test prep/3rd party resources
- ☐ Clothing for clinic/hospital duties
- ☐ Textbooks (or online copies)
- ☐ Required exams (Step 1, 2)
- ☐ Residency fees
- ☐ Social/student fees

Does your school offer any programs for additional financial support?

- ○ Yes
- ○ No
- ○ Unsure

Please describe the financial support provided by your medical school

Impact
Please rate the following statements

	Strongly disagree	Disagree	Neutral	Agree	Strongly agree
**I spent more money out of pocket during medical school than expected**	○	○	○	○	○
**The total cost of attendance listed by my medical school accurately predicted my expenses during medical school**	○	○	○	○	○
**The cost of additional resources has been a financial constraint at some point in my medical career**	○	○	○	○	○
**Financial concerns have impacted my mental health and well-being at some point in my medical career**	○	○	○	○	○

I felt like I spent ____ I expected for resources directly applicable to my medical education

- ○ Significantly less than
- ○ The same as
- ○ Significantly more than

Demographics

Age

18 — 50

Change the slider above to set a response

reset

How would you describe your gender?

- ○ Man
- ○ Woman
- ○ Non-binary/non-conforming
- ○ Other
- ○ Prefer not to answer

How would you describe your race?

- ○ American Indian or Alaska Native
- ○ Asian
- ○ Black or African American
- ○ Native Hawaiian or Pacific Islander
- ○ White
- ○ Prefer not to answer

Are you of Hispanic, Latino/a, or Spanish origin?

- ○ Yes
- ○ No
- ○ Prefer not to answer
